# RiboStreamR: a web application for quality control, analysis, and visualization of Ribo-seq data

**DOI:** 10.1186/s12864-019-5700-7

**Published:** 2019-06-06

**Authors:** Patrick Perkins, Serina Mazzoni-Putman, Anna Stepanova, Jose Alonso, Steffen Heber

**Affiliations:** 1 Bioinformatics Research Center, North Carolina State University, Raleigh, NC 27607 USA; 2 Department of Plant and Microbial Biology, North Carolina State University, Raleigh, NC 27607 USA; 3 Computer Science Department, North Carolina State University, Campus Box 8206, Raleigh, NC 27695-8206 USA

**Keywords:** Ribo-seq, Next-generation sequencing, Data analysis, Web application

## Abstract

**Background:**

Ribo-seq is a popular technique for studying translation and its regulation. A Ribo-seq experiment produces a snap-shot of the location and abundance of actively translating ribosomes within a cell’s transcriptome. In practice, Ribo-seq data analysis can be sensitive to quality issues such as read length variation, low read periodicities, and contaminations with ribosomal and transfer RNA. Various software tools for data preprocessing, quality assessment, analysis, and visualization of Ribo-seq data have been developed. However, many of these tools require considerable practical knowledge of software applications, and often multiple different tools have to be used in combination with each other.

**Results:**

We present riboStreamR, a comprehensive Ribo-seq quality control (QC) platform in the form of an R Shiny web application. RiboStreamR provides visualization and analysis tools for various Ribo-seq QC metrics, including read length distribution, read periodicity, and translational efficiency. Our platform is focused on providing a user-friendly experience, and includes various options for graphical customization, report generation, and anomaly detection within Ribo-seq datasets.

**Conclusions:**

RiboStreamR takes advantage of the vast resources provided by the R and Bioconductor environments, and utilizes the Shiny R package to ensure a high level of usability. Our goal is to develop a tool which facilitates in-depth quality assessment of Ribo-seq data by providing reference datasets and automatically highlighting quality issues and anomalies within datasets.

**Electronic supplementary material:**

The online version of this article (10.1186/s12864-019-5700-7) contains supplementary material, which is available to authorized users.

## Background

**Fig. 1:**
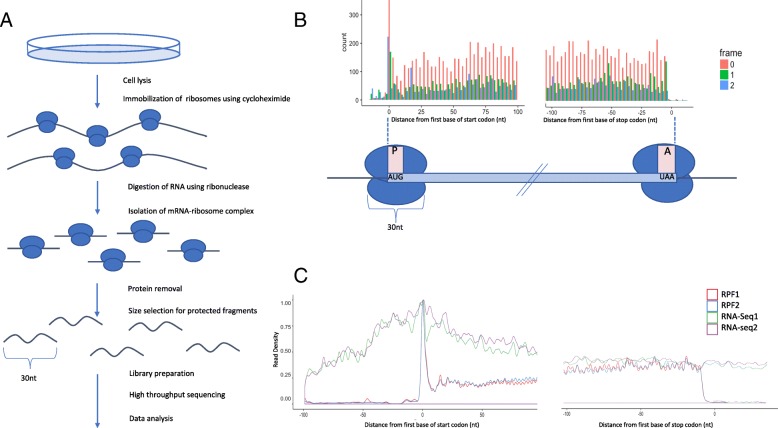
Summary of a Ribo-seq experiment and subsequent computational analysis. **a** The experiment starts with isolation of mRNA ribosome complexes, followed by nuclease digestion of mRNA sequences that are not protected by associated ribosomes. Purification of the mRNA fragments shielded by the ribosomes is then carried out, followed by library generation, deep sequencing, and data analysis. **b** Plot of ribosome protected fragment counts along the translation start and end sites. During the process of translation, the p-site holds the amino acid that is linked with the growing polypeptide chain, and therefore more accurately represents the exact position or codon within the coding sequence that the ribosome is interacting with. Because of this, an adjustment must be made to account for the positional differences between the first position of a read and the corresponding p-site of the read. Pausing of ribosomes at each codon leads to trinucleotide periodicity. The majority of reads are expected to be ‘in frame’ with the start codon. **c** Differences in Ribo-seq and RNA-seq read densities caused around the start and stop codons. In contrast to RNA-seq data, Ribo-seq data tends to show a large peak around the start codon, as well as larger percentages of sequencing reads in frame with the start codon

The rapid developments of next-generation sequencing technologies have made it possible to probe gene transcription reliably on a genome-wide level [1]. However, transcript abundance is often an insufficient proxy for protein abundance [2]. Ribosome profiling, also known as Ribo-seq, has been developed to close this gap [3, 4]. In a Ribo-seq experiment, the mRNA-ribonucleoprotein complexes formed by translating ribosomes are isolated and subjected to nuclease digestion. The mRNA fragments that are associated with ribosomes are protected from digestion and can be isolated and sequenced. Each of the resulting sequences correspond to the position of an active ribosome on the translated transcript, see Fig. 1. Typically, a Ribo-seq experiment also includes an accompanying RNA-seq component where the abundance of all transcripts is measured. Having access to both types of data allows users to estimate the number of ribosomes that are associated with an individual transcript. Hence, Ribo-seq can be used to infer high-resolution information about ribosome occupancy, translation initiation, elongation, and termination, as well as translational efficiency and translational regulation. Ribo-seq has become a popular research tool, and the amount of publicly available Ribo-seq data is rapidly increasing. Comprehensive data sets are now available for all major model organisms including yeast, bacteria, human, mouse, worm, fly, zebrafish, and Arabidopsis [3, 5–11]. The complicated experimental protocol in combination with the lack of standardization make Ribo-seq data analysis and quality control challenging tasks that consist of multiple steps and require a considerable amount of expert knowledge. Initial data pre-processing steps usually include removal of adapter sequences, low-quality reads, ribosomal RNAs, and reads that map to multiple locations. The remaining reads are then mapped to a genome or transcriptome using mapping tools such as Tophat or STAR [12, 13]. After preprocessing and mapping, a typical Ribo-seq analysis begins with multiple quality control steps, including investigating read length distributions (RLDs), assessing trinucleotide footprint periodicity, and computing read counts for various feature types, such as coding sequences, 3′ and 5′ UTRs, and non-coding RNAs. Often, meta-gene plots are computed as well. Meta-gene plots are visualizations of the aggregated read densities over a set of genes, and can aid in investigating ribosome occupancy patterns during ribosome initiation, elongation, and termination. Once a thorough quality control analysis has been performed, users often annotate translated features (ORFs, translation start and stop sites) and search for differentially translated genes. Such genes show significant changes in ribosome occupancy across different treatments, tissues, genotypes, or time-points relative to their RNA-seq levels. Additionally, Ribo-seq data can be used to investigate various aspects of translational control, such as ribosome pausing, codon usage, alternative splicing and nonsense mediated decay [14–16].

The various Ribo-seq applications have given rise to several software packages and statistical methods for data processing and quality control. For example, RiboProfiling, riboSeqR, and SystemPipeR are R packages which provide various functions for building Ribo-seq data analysis workflows and performing data QC [17–19]. Additionally, RiboGalaxy is a web based platform which hosts various standalone tools, including RiboTools, Proteoformer, plastid, and RUST, which can be used to process, analyze, and visualize Ribo-seq data [20–24]. Riboviz is another tool which allows browser based comparative analyses of published ribosome-profiling datasets [25]. See [26] for a detailed review of computation resources for ribosome profiling. A comprehensive Ribo-seq analysis can typically involve multiple of these software packages, and often requires a considerable amount of expert knowledge, as well as experience in programming or command line usage. Hence, there is a distinct need for a flexible and user-friendly environment for comprehensive Ribo-seq analyses which is accessible to mainstream biologists who lack training in bioinformatics. The goal of the riboStreamR platform is to provide such an environment. RiboStreamR is a web application written in R that hosts a suite of custom tools for Ribo-seq data analysis. These tools aid in data processing, quality control, and visualization of Ribo-seq and RNA-seq data, and facilitate user customization and reproducibility.

The rest of this paper is organized as follows: the implementation section discusses the design of the riboStreamR environment, including data processing steps and platform design. Next, the results section discusses the basic functionality and performance of riboStreamR, including graphic customization and anomaly detection. In the discussion section, we describe advantages and shortcomings of our web platform, as well as our planned future work. A worked example of a riboStreamR data quality analysis is also provided in Additional file 1.

## Implementation

### Environment

RiboStreamR is publicly available as a web application. We have chosen R and Bioconductor as the basis for our implementation. R is an open source and extremely popular programming language for applications in the field of bioinformatics. The Bioconductor project within R provides various previously established functions and packages for next generation sequencing (NGS) data analysis [27]. In addition, it also offers vast options for customization of graphics and visualizations, and it provides easy access to a wealth of statistical and machine learning tools. The R code for the application is available at https://github.com/pjperki2/riboStreamR.

In order to make riboStreamR an accessible and user-friendly web application, the Shiny package was used. Shiny is an R package which supports the development of interactive web applications by converting R code into CSS and HTML. The web interface of riboStreamR gives the user the ability to interact with their data via a streamlined graphical user interface instead of a command line or programming language. This supports our aim to make data analysis more manageable for researchers that lack experience in programming. Additionally, a server-based platform places the computational load on the host server instead of the user’s computer and removes the burden of the user having to download and install multiple different pieces of software or R packages.

### Data processing and platform design

**Fig. 2:**
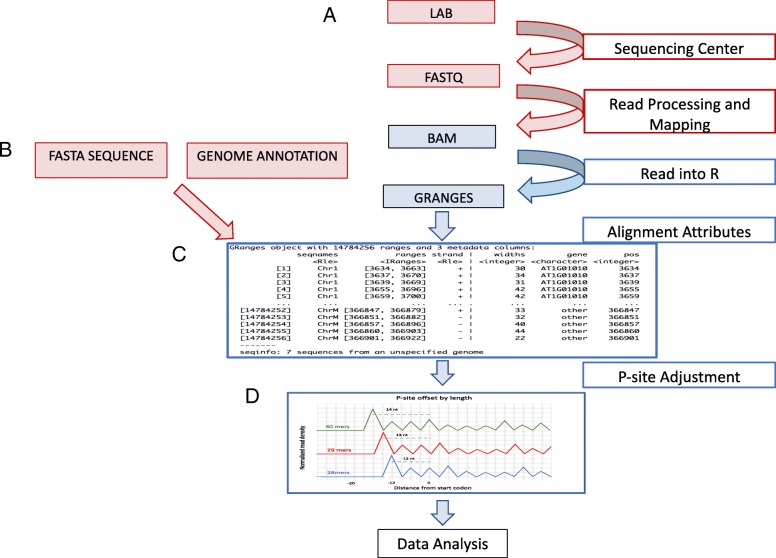
RiboStreamR Data Processing Pipeline. **a** Overview of flow of data from sample collection within a lab to processing in R. The red boxes and arrows are steps which are not handled by riboStreamR, while those in blue are handled within riboStreamR. **b** RiboStreamR requires input of three file types: a set of Bam files, a genome annotation file, and a fasta file containing the genome nucleotide sequences. **c** The uploaded sequencing data are converted into a GRanges object, where each row is an individual alignment, and every column contains attribute information (metadata) about the alignment. **d** P-site adjustment method. Reads are separated by length and a meta-gene read density plot around the translation start sites is produced for each read length. The p-site adjustment for each respective alignment length is chosen to be the distance from the largest in-frame upstream peak to the translation start site

An analysis with riboStreamR begins at the ‘Data Upload and Preprocessing’ page, where the user uploads sets of aligned reads (.bam files), as well as a genome sequence file (.fasta file), and a reference genome annotation file (.gff3 or .gtf file). The flow of data in a typical Ribo-seq experiment is depicted in Fig. 2. BAM files for both Ribo-seq and RNA-seq data are supported. Each BAM file is read into the R environment as a distinct GRanges objects, from the GenomicRanges package [28], while multiple BAM files can be combined and represented as a GRangesList object. When initially generated, these matrix-like objects contain information on each individual alignment in the BAM files, including the chromosome, strand, and start/end position. A set of additional descriptive attributes (see Table 1) are then computed for each alignment. The attributes are used to dynamically filter and subset the data in order to increase graphical customization. Subsequently, p-site computation and adjustment is performed for each Ribo-seq read. The p-site is the position at which ribosomes process codons, and, as compared to the start or end position of the read, is a more accurate approximation of the specific site where the ribosome is interacting with the mRNA molecule [11]. This adjusted position can then be used to determine whether the ribosome is ‘in-frame’ with a corresponding start codon. Figure 2d shows an example how the p-site is inferred for various read lengths.

**Table 1:** Description of alignment attributes. Each attribute is contained within a separate metadata column in the GRanges object

Attribute Name	Description
seqnames	Chromosome on which the aligned read is mapped.
ranges	Start and end position of the alignment in genomic coordinates.
strand	Strand to which the aligned read is mapped.
sample	Sample name from which the alignment originates.
exp	Experiment type of the sample from which the alignment originates, either ‘Ribo’ for Ribo-seq, or ‘RNA’ for RNA-seq.
length	Length of the aligned read in nucleotides.
gene	Gene to which the read is mapped. Corresponds to Gene IDs within the provided annotation file. ‘Other’ if not mapped within a gene.
feature	Feature type to which the read is mapped. Feature types correspond to those included in the user-provided annotation file.
pos	Genomic position of alignment based on p-site adjustment.
start_dist	Distance from transcription start site (TSS) of a gene to p-site position, in transcript coordinates (with introns removed). The major isoform of each gene is used to calculate this distance.
end_dist	Distance from p-site position to translation stop codon (TSC) of a gene, in transcript coordinates (with introns removed). The major isoform of each gene is used to calculate this distance.
gc	The percentage of nucleotides in the aligned read which are G’s or C’s.
mapq	The mapq score of the alignment. Typically, alignments with a mapq score of 50 are considered uniquely mapped, while all other scores are considered multi-mapping.
frame	The trinucleotide frame of a read’s p-site, relative to the TSS of the gene’s major isoform. Reads that map within an mRNA are assigned either a 0, 1, or 2, while reads which map outside the mRNA are assigned ‘none’.

Once data pre-processing is completed for all BAM files, the analysis proceeds with downstream tools which perform quality control, analysis, and visualizations of the processed data. All tools in our platform are essentially standalone applications, and therefore the user may choose to use them in any order.

## Results

### Functionality

**Fig. 3:**
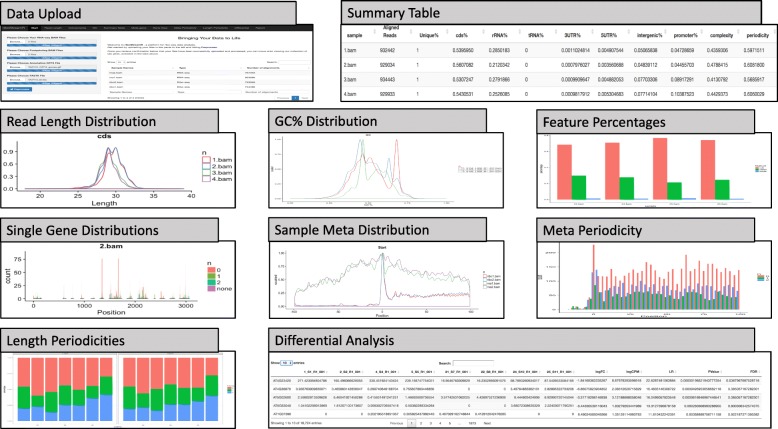
Output examples for each tool in the platform. Descriptions of each tool can be found in Table 2

RiboStreamR includes 10 individual tools which facilitate quality control, downstream analysis, and result visualization. The individual tools can be accessed through tabs across the top of the application. Each tool consists of a toolbar, where graphical parameters can be adjusted, and an output pane, which displays the graphical output of the tool. A ‘Submit’ button at the bottom of each toolbar generates output for the selected set of parameters; see Fig. 3 for an example. The tools are described in Table 2 and depicted in Fig. 3. The platform is not structured as a step-by-step pipeline, but as a set of standalone tools which can all be accessed once data input and processing is complete. While facilitating a comprehensive evaluation of Ribo-seq data quality is the main intention of the platform, riboStreamR also supports the analysis of RNA-seq experiments, including differential expression analyses. Our tools use several previously established R packages for NGS applications including GenomicRanges, GenomicFeatures, and GenomicAlignments for the storage and manipulation of the alignments; ShortReads, systemPipeR, BioStrings, riboSeqR, and RSamTools for the processing of the alignments; edgeR for inferring differentially translated genes, and ggplot2, cowplot, and grid for producing the wide range of different visualizations [28–33]. A case study, which demonstrates the use of the platform’s tools within the context of a typical Ribo-seq quality analysis has been provided in Additional file 1.

**Table 2:** Description of Tools within riboStreamR

Tool Name	Description
Data Upload	The tool allows users to upload their RNA-seq, Ribo-seq, annotation, and fasta files. After data upload and pre-processing, it displays a table with the number of alignments within each of the uploaded BAM files.
Summary Table	The tool provides a table with summary statistics for each sample, including total number of alignments, percentage of uniquely mapped reads, feature percentages, complexity, duplication values, and periodicities.
Read Length	Computes read length distributions for any combination of input files or data subsets.
GC %	Computes GC percentage distributions for any combination of input files or data subsets.
Feature %	Generates bar charts of the relative numbers of alignments mapping to the different feature types or any other alignment attribute.
Single Gene	Visualizes the read densities within single genes. Genes are displayed in genomic coordinates with gray regions indicating exons. Density bars can be color coded to differentiate between data subsets.
Meta Periodicity	The tool generates a meta gene distribution around the TSS and TSC for a single sample, showing the read density at each nucleotide. This is useful for gauging the level of periodicity.
Sample Meta Distribution	Generates line graphs of the distribution of reads around the TSS and stop codon. The tool allows users to compare multiple samples or other read subsets.
Length Periodicity	Computes a bar graph that shows the relative number of alignments within each frame.
Differential Analysis	The tool computes a table which contains RPKM values for each gene, as well as the results of a differential analysis using edgeR, including the logFC, *P*-value, and FDR. Can perform differential expression or translation analyses.
Report Generator	The tool generates a report containing the outputs of the user selected tools, as well as the parameters used to produce the output, a description of the processing methods, and any user-provided notes.

### Graphic customization

**Fig. 4:**
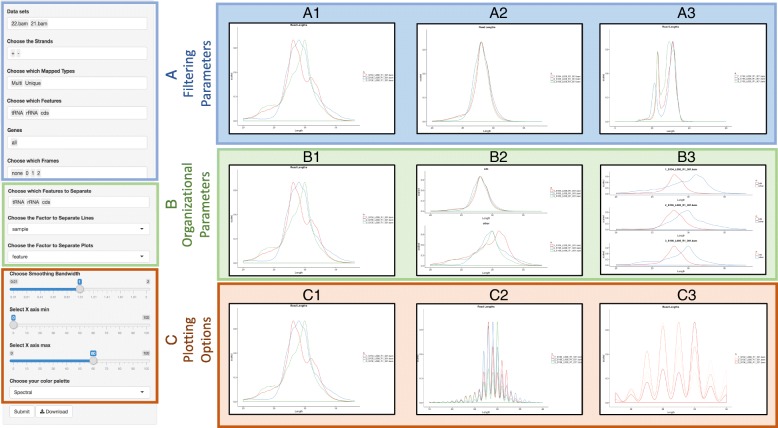
Examples of riboStreamR’s graphical output customization options. On the left side is a representation of the toolbar. (A) Filtering parameters are shown in blue, and allow plotting of distinct subsets of the input data; (A1) Read Length Distribution (RLD) plot where each line represents the alignments from 3 different samples; (A2) RLD plot where only alignments mapped to the CDS are included; (A3) RLD where only reads mapping to tRNA or rRNA regions are included. (B) Organizational parameters, shown in green, allow the user to adjust how the filtered data are grouped and positioned in the output; (B1) Same as A1; (B2) RLD plots where the two plots separate between alignments mapped within a CDS and those mapped to any other feature, and the lines separate between three different samples; (B3) RLD plots where each plot is a separate sample, and the two separate lines represent reads mapping to different feature types. (C) Examples of different plotting parameters, shown in orange, which change the aesthetics of the graphical output; (C1) same as A1 and B1; (C2) reduced bandwidth of line plot to simplify the comparisons between each separate read length; (C3) reduced range of x-axis range, as well as different color scheme of plots to highlight differences between samples

RiboStreamR facilitates graphical customization via various options for plot aesthetic and layout, as well as through dynamic filtering and sub-setting of data. Visualizations are customized through the use of the toolbar, which is located on the left side or bottom of each tool. The toolbar has three different types of adjustable parameters, see Fig. 4 for examples. *Filtering parameters* allow users to select the alignments they want to include in their analysis; alignments can be filtered by any of the attributes listed in Table 1. *Organizational parameters* change how the selected alignments are grouped and positioned within the output. For example, users may wish to compare the alignments from one sample against alignments from all other samples combined, or they might want to compare subsets across a specific set of attributes, such as feature type, read length, and GC content. *Plotting parameters* affect the appearance or dimension of the output. Examples include adjustable axis values, axis labels, color schemes, and line types. Through the adjustment of these three types of customization parameters, users are able to create presentation quality graphics which are specific to their exact experimental inquiries. The output from each individual tool is also downloadable as a PDF image.

### Reference datasets and anomaly detection

RiboStreamR allows users to integrate a set of high-quality reference datasets, together with their own data, into their analyses. The reference datasets were collected from Arabidopsis roots and shoots, and were generated using a protocol which yields high-resolution and high-quality Ribo-seq data [11]. The reference datasets can be chosen as an input option from the data upload page, and included as a sample in any of the downstream tools. In addition, these datasets can be used as references for anomaly detection. One of the goals of our platform is to identify abnormal datasets. We have implemented four independent anomaly detection strategies with the Summary Table tool:

1. Anomaly detection based on expert defined thresholds for read periodicity, and percent of reads mapped to rRNA, tRNA, and CDS regions;
2. Outlier detection based on interquartile ranges derived from user samples, using Tukey’s fence [34].
3. Outlier detection based on user-selected controls. We compute summary QC metrics (including periodicity, feature percentages, and percentage of uniquely mapped reads) from the selected controls, and compare them to the corresponding values derived from the user’s samples. Outliers are defined using percent error calculation, with a threshold of 25% difference [35].
4. Outlier detection based on our supplied reference data sets, using the method describe in item 2 above. We have precomputed a large set of quality metrics for various analysis parameter configurations to facilitate fast and accurate comparisons between the user-samples and our reference data.

Suspicious values found using these methods are flagged and reported in a separate table within the output pane.

### Report generation

The Report Generation tool within riboStreamR produces a comprehensive R Markdown report of the performed quality control and data analysis results. This tool can run and summarize a full analysis in one click, or it can be used to combine the output of any number of the individual tools into one PDF or HTML document. Textual notes or summaries which describe the processing or analysis steps may also be appended to each graphic. Based on the chosen configuration, the generated reports include important information about the selected analysis parameters, as well as user-provided text as notes, figure summaries, or bibliographies for inclusion in publications. Additionally, individual graphics generated from each tool maybe downloaded independently within each tool for incorporation in publications or research papers.

### Performance

A complete Ribo-seq analysis using riboSeqR typically takes around 30 min per sample, where we assume that the underlying BAM file contains around 20 million reads. This time does not include fastq file processing and read mapping steps, which are not handled by our platform at this time. The main performance bottlenecks in the riboStreamR framework are data upload, data pre-processing, and output generation. The BAM file will be read into R as a 400 MB initial GRanges object approximately. The process of uploading data from the user’s machine to the web server took us approximately 15 min per sample. Once the data are uploaded, the pre-processing steps, which include computing alignment attributes and p-site adjustments, typically take between 10 and 15 min per sample. The additional alignment attributes computed during preprocessing typically result in a about five-fold increase in the size of the initial GRanges objects. Finally, the time it takes to generate output graphics is usually less than one minute per sample and tool. Certain tools, including the summary table and the meta distribution plots, utilize an alignment subsampling strategy in order to reduce the time it takes to produce output even further.

## Discussion

### Need for a web application based Ribo-seq analysis platform

As described above, various tools for streamlining quality control, analysis, and visualization of Ribo-seq data exist. Although extremely useful, the environments, user-interfaces, and infrastructures, of these tools vary considerably, and some of them require the knowledge of command line usage, R, or some other programming language. There exists a need for a platform which provides a consolidated suite of analysis tools that are accessible through a user-friendly GUI. The RiboGalaxy [20] server is a tool that addresses some of these pain points. However, despite its popularity and usefulness, RiboGalaxy relies on combining processing steps and outputs from various third party tools, and does not focus on consistency and compatibility amongst the different tools. Our riboStreamR application is designed to fill this gap. The platform has the advantage of being an open-ended environment in which it is not a requirement for the user to follow a specific step-by-step procedure to ensure output/input compatibility. RiboStreamR takes advantage of the notable flexibility and functionality that R provides, but in a manner which removes the need for users to have programming experience in R, or another programming language. Of course, there are also certain advantages of alternative software tools over riboStreamR. For example, providing more upstream tools, such as fastq file QC; supporting a wider range of QC metrics, such as codon density analysis; functionality to map footprints to a reference genomes; and supporting integration with command line tools or other NGS software.

### Future work

We plan to expand the utility of riboStreamR as a quality control and data analysis platform. As riboStreamR is still currently in development, there are many prospective features for use in both upstream processing and downstream analysis which are not yet fully implemented. These include additional tools for fastq quality analyses, optimized algorithms to map fastq files of ribosomal footprints to a reference genome, and additional downstream analysis tools to investigate codon usage, and to identify ribosome pause-sites and functional uORFs. Currently our tool has primarily been tested using data from *Arabidopsis thaliana*, but our goal is to perform extensive validation experiments using data from other species and to include corresponding datasets as references. A further research goal is the development and integration of more robust anomaly detection algorithms tailored for Ribo-seq data. We also plan to expand riboStreamR’s use of automation to simplify user’s analyses. Examples include the automatic generation of textual summaries and the automatic optimization of plotting parameters. Lastly, we plan to further optimize the platform’s underlying R code, for example by compressing sequence attributes to decrease memory consumption.

## Conclusion

The riboStreamR platform aims to harness the wealth of resources provided by the R and Bioconductor universe within a user-friendly web application. In contrast to the pipeline infrastructure of many NGS analysis systems, riboStreamR is designed as a suite of tools, where each tool is available to the user once their data have been uploaded and preprocessed. RiboStreamR is designed around an easy-to-use GUI which provides users with various options for graphical customization via dynamic filtering and arranging of data. This more accessible environment eases many of the burdens that typically make Ribo-seq data analysis a daunting task. Further improvements of platform automation and anomaly detection algorithms are priorities for future work.

## Availability and requirements

**Project name:** *riboStreamR Ribo-seq analysis platform.*

**Project home page:** http://uhura.cos.ncsu.edu:3842/

**Archived version:** *riboStreamR.*

**Operating system(s):** Platform independent.

**Programming language:** R.

**Other requirements:** R version ≥3.2, Bioconductor version ≥3.2.

**License:** Artistic-2-0.

**Any restrictions to use by non-academics:** none.

## Additional files

Additional file 1: Case study A case study demonstrating the functionality of the various tools in riboStreamR for QC analysis. (DOCX 80850 kb)

## Abbreviations

*BAM*: Binary version of sequence alignment map format
CSS: Cascading Style Sheets is a style sheet language used for describing the presentation of a document written in a markup language
*FASTQ*: short read sequence file format
HTML: Hypertext Markup Language is the standard markup language for creating web pages and web applications
*NGS*: Next generation sequencing
PDF: Portable document format
QC: Quality control
*Ribo-Seq*: NGS profiling of mRNA populations bound to ribosomes
RLD: Read length distribution
*RNA-Seq*: NGS profiling of mRNA
*SAM*: Sequence alignment map format
TSC: Translation stop coon
TSS: Translation start site
UTR: Untranslated region
