# Supplementary material for: RiboStreamR: a web application for quality control, analysis, and visualization of Ribo-seq data
Source: BMC Genomics. 2019 Jun 6;20(Suppl 5):422. doi: 10.1186/s12864-019-5700-7 (PMC6551240; doi:10.1186/s12864-019-5700-7)
Supplement: Supplementary file 1 — Case study A case study demonstrating the functionality of the various tools in riboStreamR for QC analysis. (DOCX 80850 kb) [file 12864_2019_5700_MOESM1_ESM.docx]

Additional File 1

**Case Study Introduction**

The following case study illustrates the functionality of riboStreamR by guiding the reader through a basic example of a Ribo-seq quality analysis. In this study, we will employ the use of riboStreamR’s various QC tools to investigate the quality of four different samples from [1], each of which differ in treatment/condition. The data can be found at <https://www.ncbi.nlm.nih.gov/sra/?term=SRP056795>. A reference sample from [2] is also included, which can be found at <https://www.ncbi.nlm.nih.gov/geo/query/acc.cgi?acc=GSE81332>. Table S1 below provides the file names used in the following analysis and the corresponding sample names from their data repositories.

Table S1. File names along with their corresponding repository sample names.

| **File name** | **Sample name** |
| --- | --- |
| rpf_air_wt_1 | Col-Air-RFP1 |
| rpf_air_upf2_1 | Upf2-Air-RFP1 |
| rpf_ehtylene_wt_1 | Upf2-Air-RFP1 |
| rpf_ethylene_upf2_1 | Upf2-Ethylene-RFP1 |
| Riboseq_root_2_1 (reference) | Riboseq_root_2 |

All of the samples were subjected to adapter trimming with the FASTX toolkit (<http://hannonlab.cshl.edu/fastx_toolkit/>), and subsequently mapped with Tophat, using default mapping parameters [3]. TAIR 10 annotation and fasta files were used for the mapping [4].

**Data upload and preprocessing**

The first step is to upload and preprocess the data, which we can do within the **Start** tab, shown in Figure S1. Since we are only dealing with Ribo-seq data, and are not using corresponding RNA-seq data, all we need to do is upload our Ribo-seq BAM files and our Arabidopsis annotation file in the toolbar to the left. We can also choose the reference file riboseq_root_2_1 from the Please choose reference RPF file upload box. Once this has been done, we can click Preprocess at the bottom of the toolbar to initialize data preprocessing. This is the most rate-limiting step within the riboStreamR platform. Once preprocessing is complete, a table will appear in the output pane of the **Start** tool which lists the samples which were uploaded, as well as the number of reads in each sample. At this point, we are ready to move to our downstream QC tools.

Figure S1. Start tab, showing upload of all 5 files, and the resulting table with experiment type and read count information.

**Read length distributions**

The **Read Length Distribution** tool allows us to visually inspect our data for read length anomalies. Ribosomes typically shield mRNA fragments of around 30 nucleotides (nt) in length. Therefore, most the reads in high quality Ribo-seq datasets are between around 25 and 32 nucleotides in length. An abundance of reads outside of this range often indicates issues in some preparation or processing step.

In order to produce read length distributions for each sample plotted on one set of axes, we first make sure all our data sets are included in the Data sets parameter at the top of the toolbar, and then we set the Choose the factors to separate plots parameter to ‘none’. We can then hit Submit button, and the plot shown in Figure S2 is generated, where each line represents all the reads in an individual sample. To further inspect the read length distributions, we can create separate plots for different feature types. Figure S3 is produced by changing the Choose the factors to separate plots parameter to ‘features’, and setting the Choose which features parameter to include ‘cds’, ‘rRNA’, and ‘tRNA’. This functionality is useful for investigating the source of any irregular read lengths.


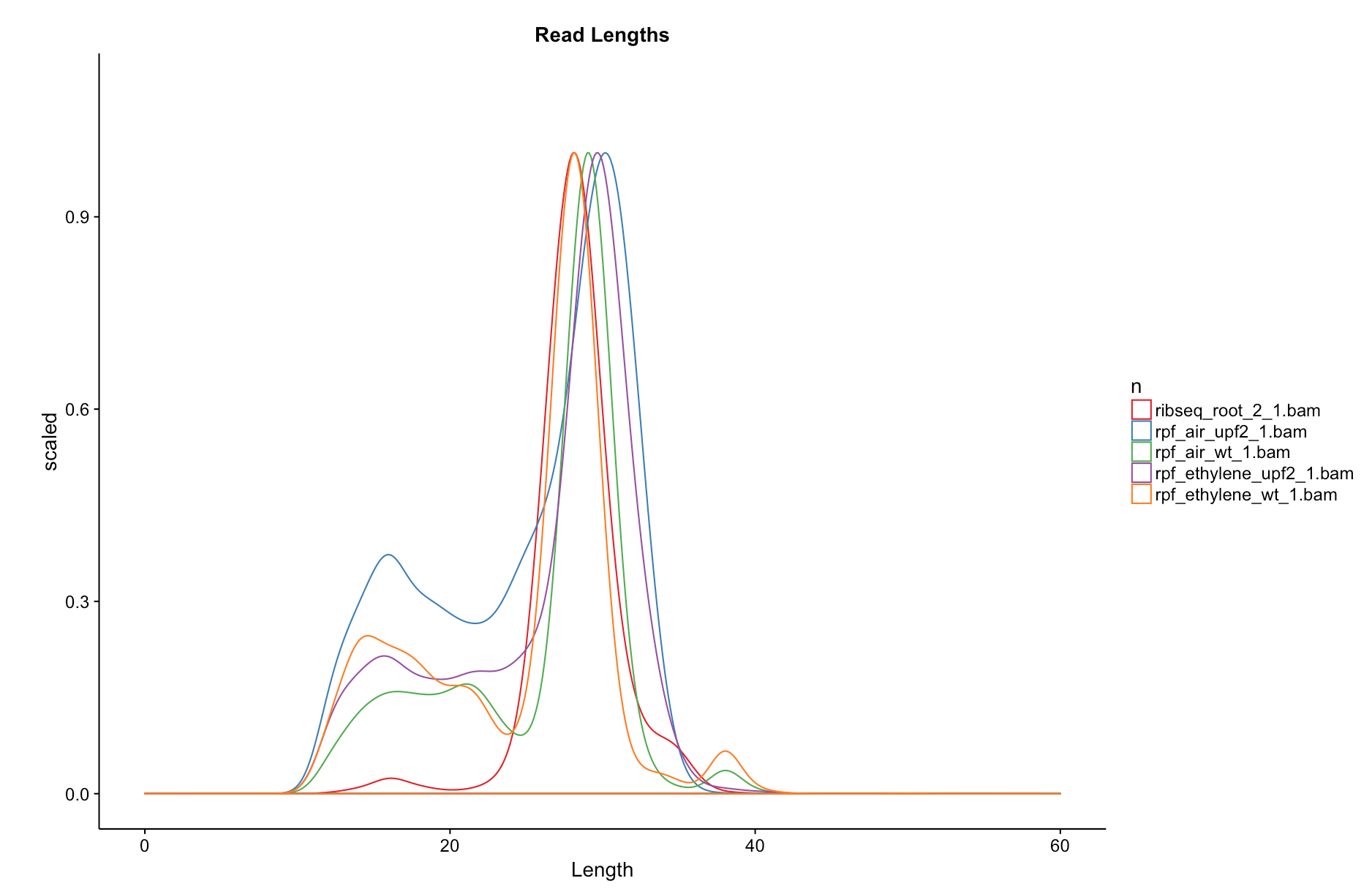


Figure S2. Read length distributions of all the reads in each of the six samples, displayed in one

plot.


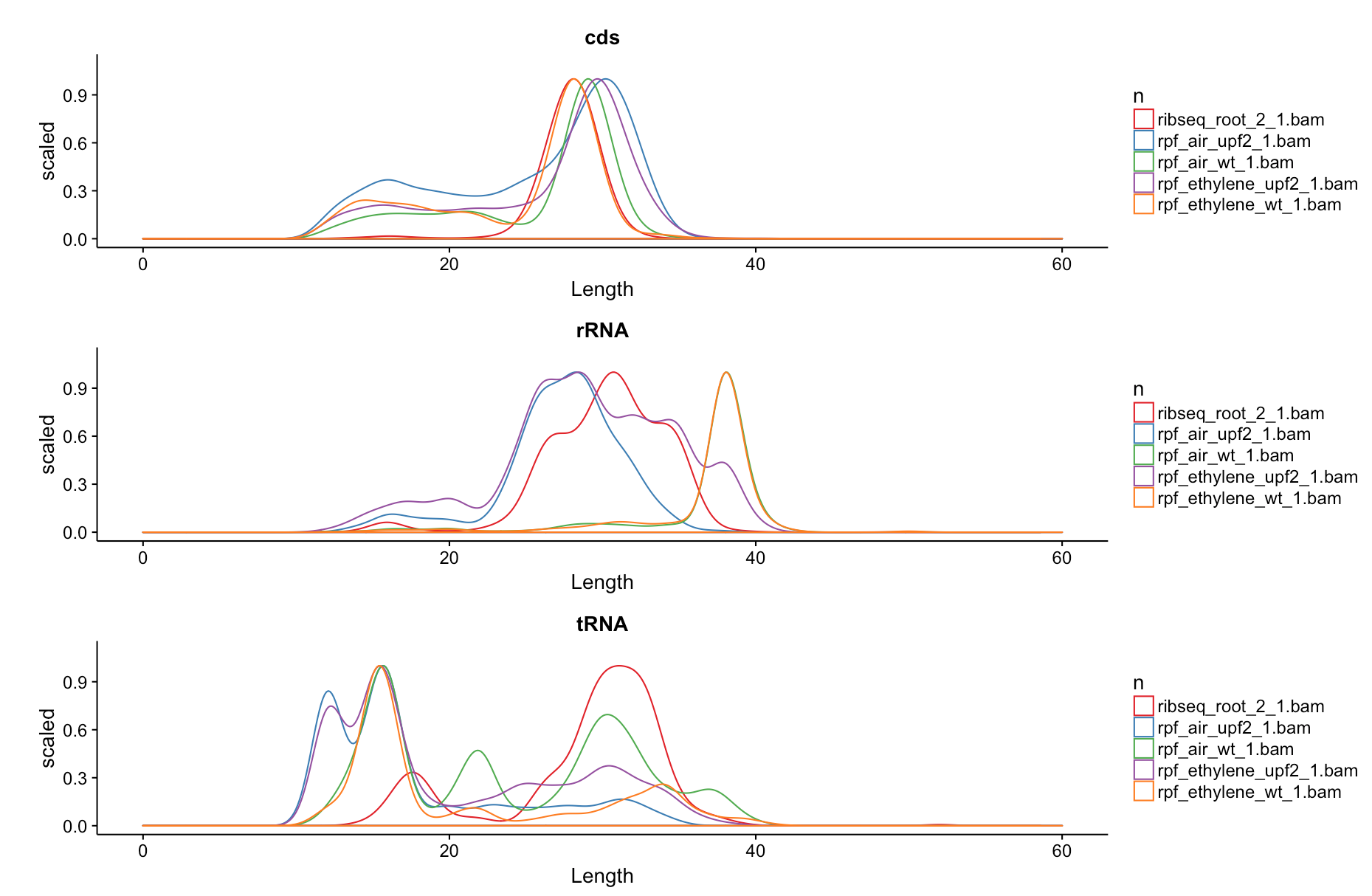


Figure S3. Read length distributions with plots separated by three feature types, ‘cds’, ‘rRNA’, and ‘tRNA’.

**Feature percentages**

The **Feature Percentages** tool can be used to inspect what fraction of the reads in each sample have been mapped to the different feature type, such as coding sequences (cds), rRNA, tRNA, 5’ and 3’UTRs, and intergenic regions. It is often important to look at the relative abundances of reads mapping to the coding sequence, rRNA, and tRNA regions, as tRNA and rRNA contamination are some of biggest issues in riboseq QC. This can be done by selecting ‘cds’, ‘tRNA’, and ‘rRNA’ from the Choose which features parameter, and hitting Submit*.* This yields Figure S4, in which we compare the relative abundance of reads mapping to each feature between the samples. To further investigate the relationship between the read length and the mapped feature type, we can change the Choose the factor to separate groups parameter from ‘none’ to ‘sample’, set the Choose the factor to separate bars parameter to ‘width’, and the Choose read length range parameter to 20:30. Additionally, by selecting all the feature types in the Choose which features parameter field, and leaving the Choose which features to separate parameter as ‘cds’, ‘tRNA’, and ‘rRNA’, we create another feature type in our plot called ‘other’, which represents all of the reads mapping to features other than cds, tRNA, and rRNA. Submitting these parameters produces Figure S5.

Figure S4. Feature percentages plot for cds, rRNA, and tRNA, where each group of bars represents an individual sample. The bars report relative abundance between the feature types, and therefore the percentages within each sample always add up to 1.

Figure S5. Feature percentages plot, where each group of bars is a sample, each bar is a read length, and each color is a feature type. The ‘other’ feature type represents reads mapping to any feature which is not coding sequence, tRNA, or rRNA.

**GC percentages**

In order to investigate potential GC biases caused by the different treatment/conditions, we will use the **GC Percentages** tool to look at the relative GC ratio amongst the reads in the samples. Figure S6 is produced by using the default parameter sets within the tool. We can also inspect to see if there is a significant difference between the GC% in uniquely-mapping and multi-mapping reads by setting the Choose the factor to separate plots parameter to ‘mapping’, shown in Figure S7.


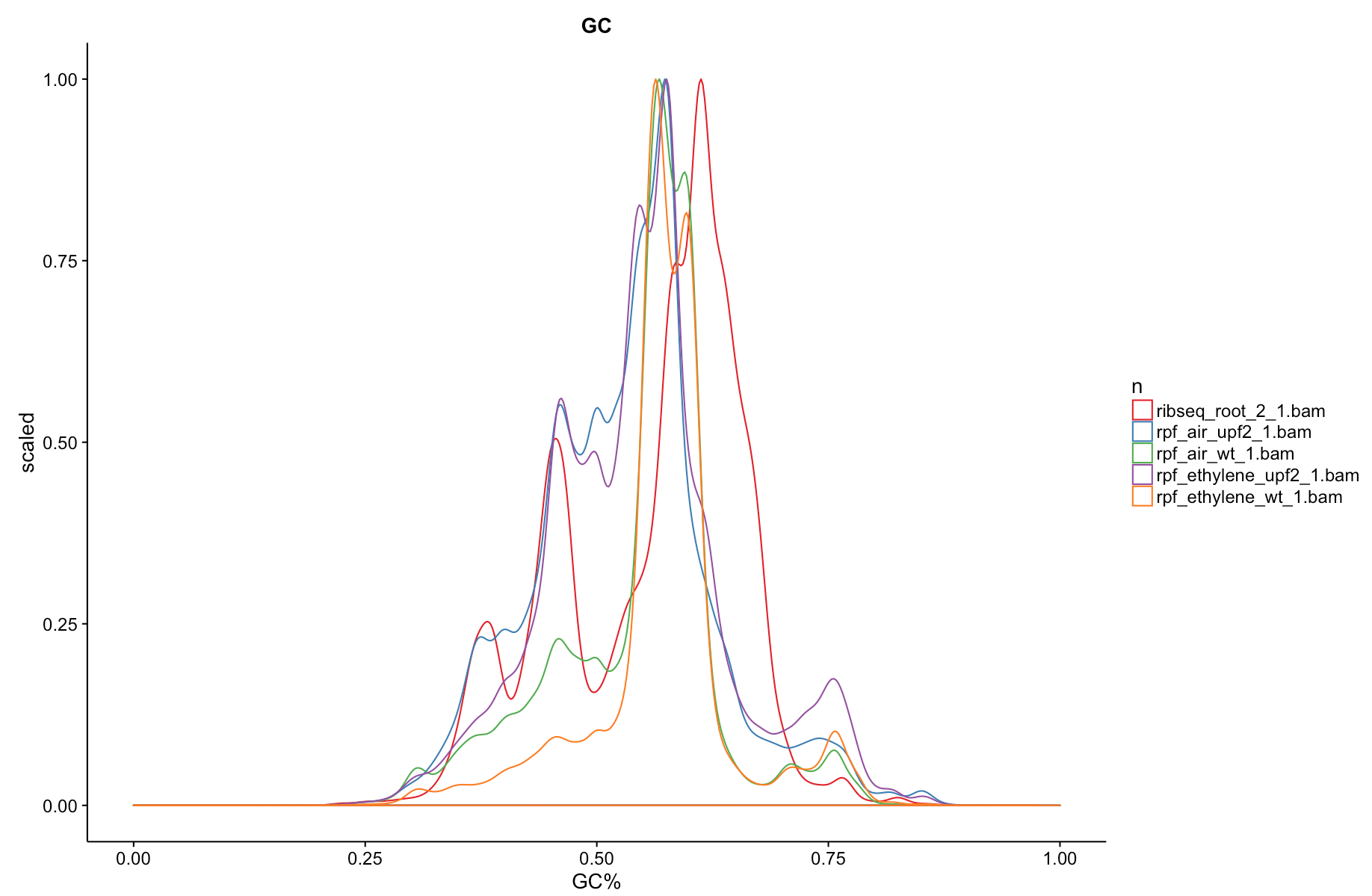


Figure S6. GC percentages plot, with a separate line for each sample.


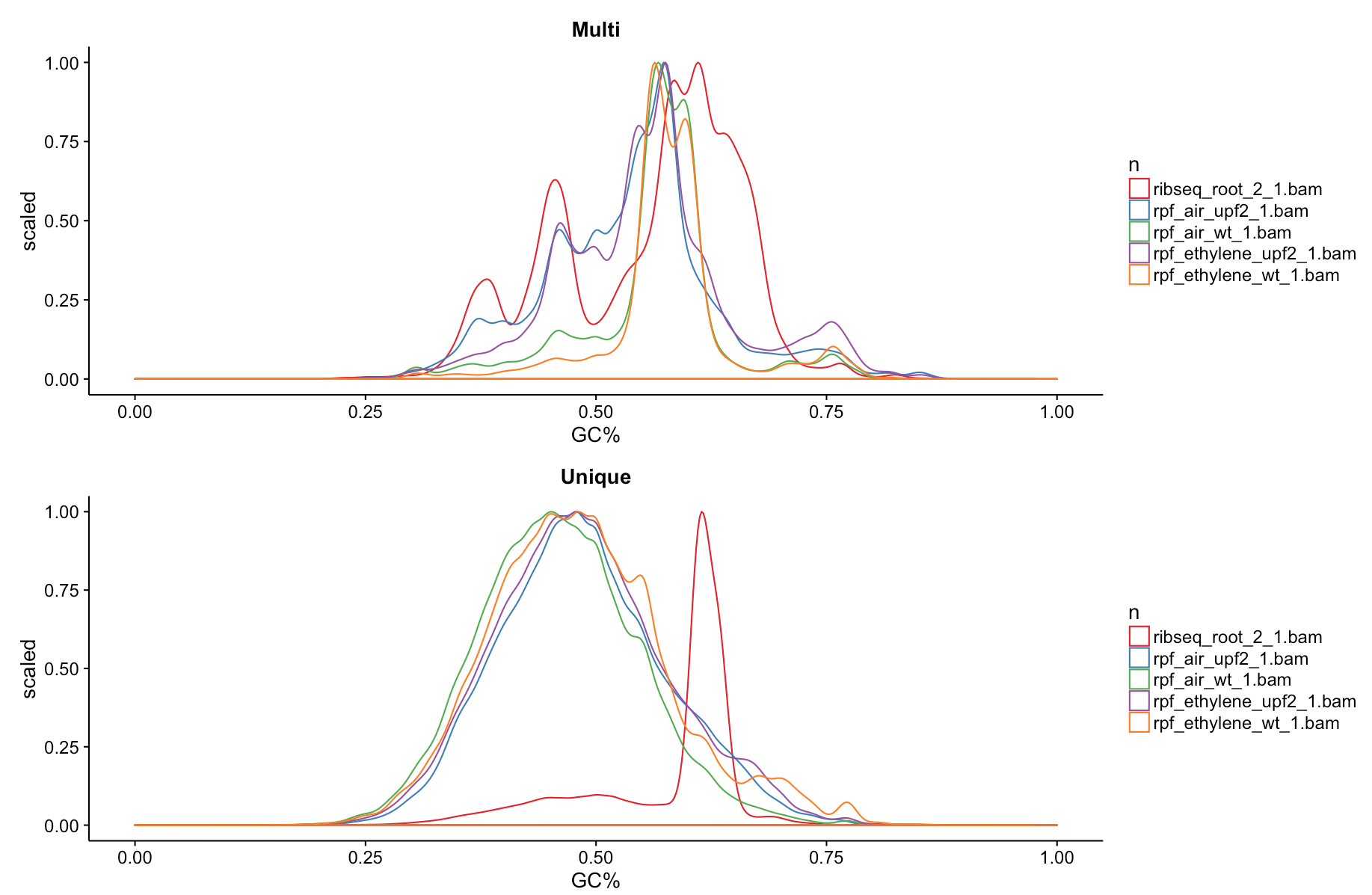


Figure S7. GC percentages plot, where the plots have been separated by mapping types.

**Summary tables**

The **Summary Table** tool displays summary QC metrics for each included sample. This includes metrics such as percentage of reads which are uniquely mapped, percentages of reads which map to each feature type, trinucleotide periodicity values (% of reads mapping in the major frame, the higher the better), and complexity values (% of reads mapping to a unique location, the higher the better). Figure S8 shows a summary table comparing the samples using the default parameter settings. This tool uses sub-sampling of 1 million reads from each sample to reduce computation time.


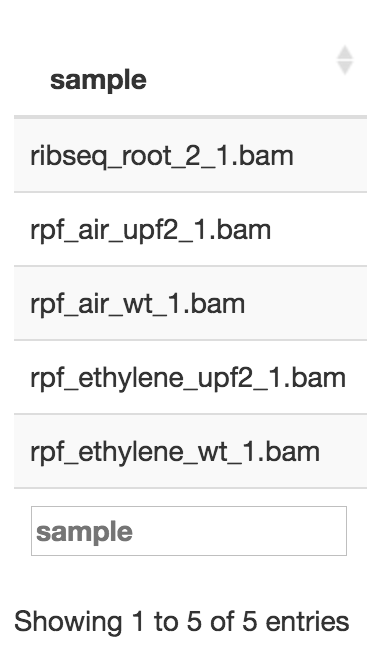

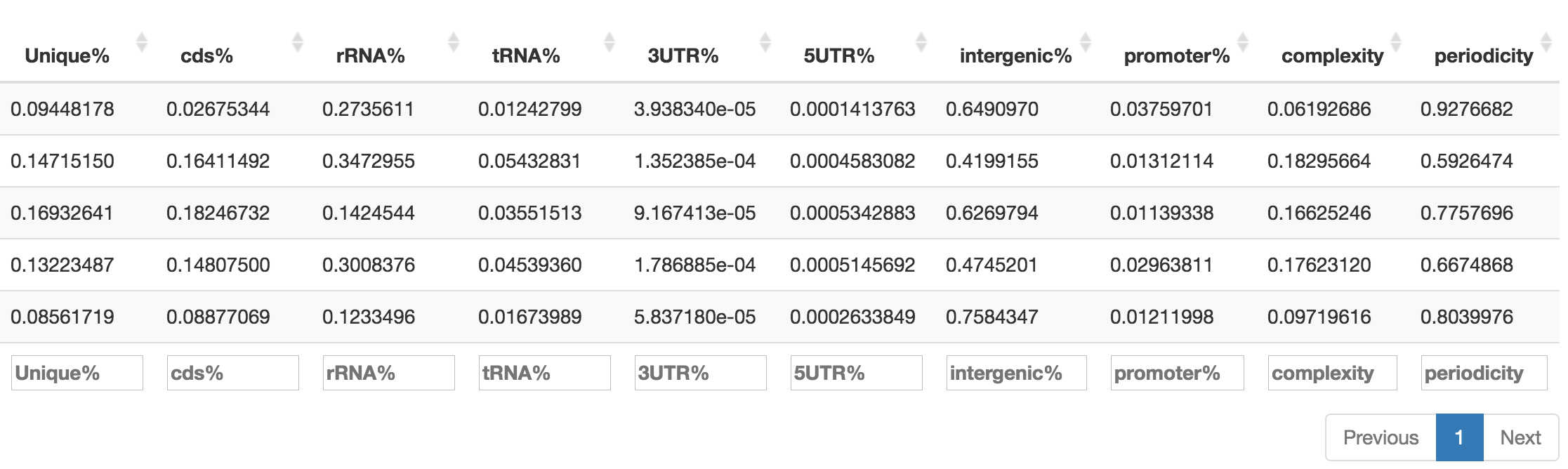

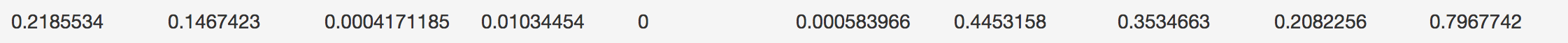


Figure S8. Summary QC metric table for the 5 samples.

**Sample meta distributions**

Meta-gene plots are visualizations of the aggregated read densities over a set of genes. Meta-gene plots aid in investigating read occupancy patterns during ribosome initiation, elongation, and termination. The presence of distinct read distribution peaks around the start and stop codons in genes, as well as minimal read occupancy in the 5’ and 3’ UTRs, are typical indicators high-quality data. The **Sample Meta Distribution** tool produces line graphs showing the aggregated distributions of reads around the start and stop codons for all coding sequences. Each read is assigned to a specific location corresponding to its approximated p-site. This tool is useful for comparing the meta-gene distributions of multiple samples. Figure S9 employs the default parameter set for this tool and produces separate plots around the start and stop codons, with a line for each sample.


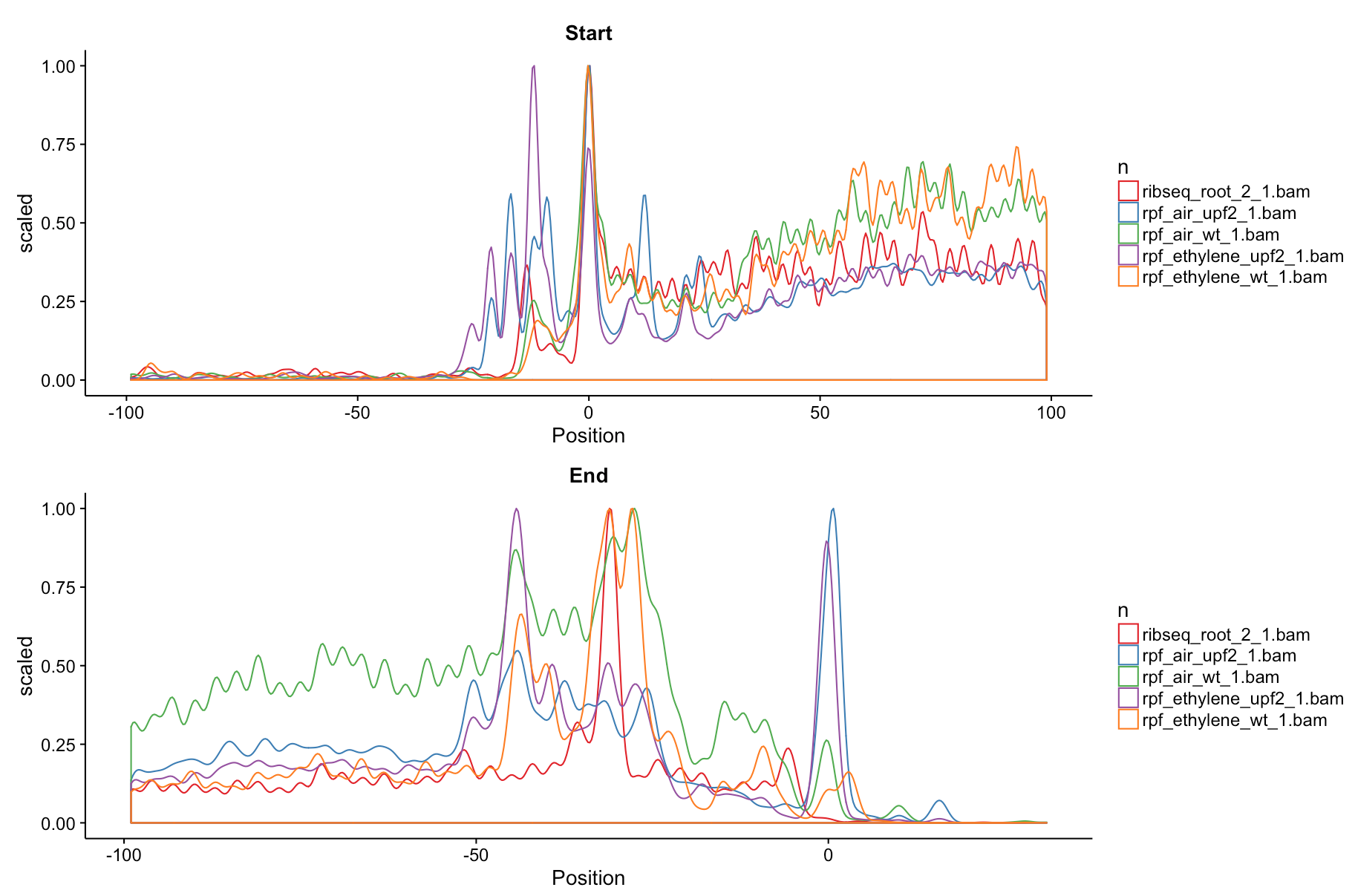


Figure S9. Sample meta gene distributions, in which each sample is represented by its own line. The two separate plots represent the aggregated read density across all genes at the start and end of the coding sequence.

**Meta-gene periodicities**

The **Meta-Gene Periodicity** tool also produces meta gene plots, but they are displayed as histograms rather than line graphs. This allows for read occupancy resolution at the nucleotide level, and allows us to inspect the periodicity levels of each sample. The only drawback is that only one sample may be plotted per set of axes, making comparisons between samples less convenient. Figure S10 shows meta-gene periodicity output when the Choose factor to separate plots parameter is set to ‘sample’. The different colored bars represent reads in each of the different frames. Reads within the frame ‘none’ are those which do not map within the coding sequence. High periodicity is indicated by a large abundance of reads in frame 0 (also called the ‘major frame’) relative to the other frames.


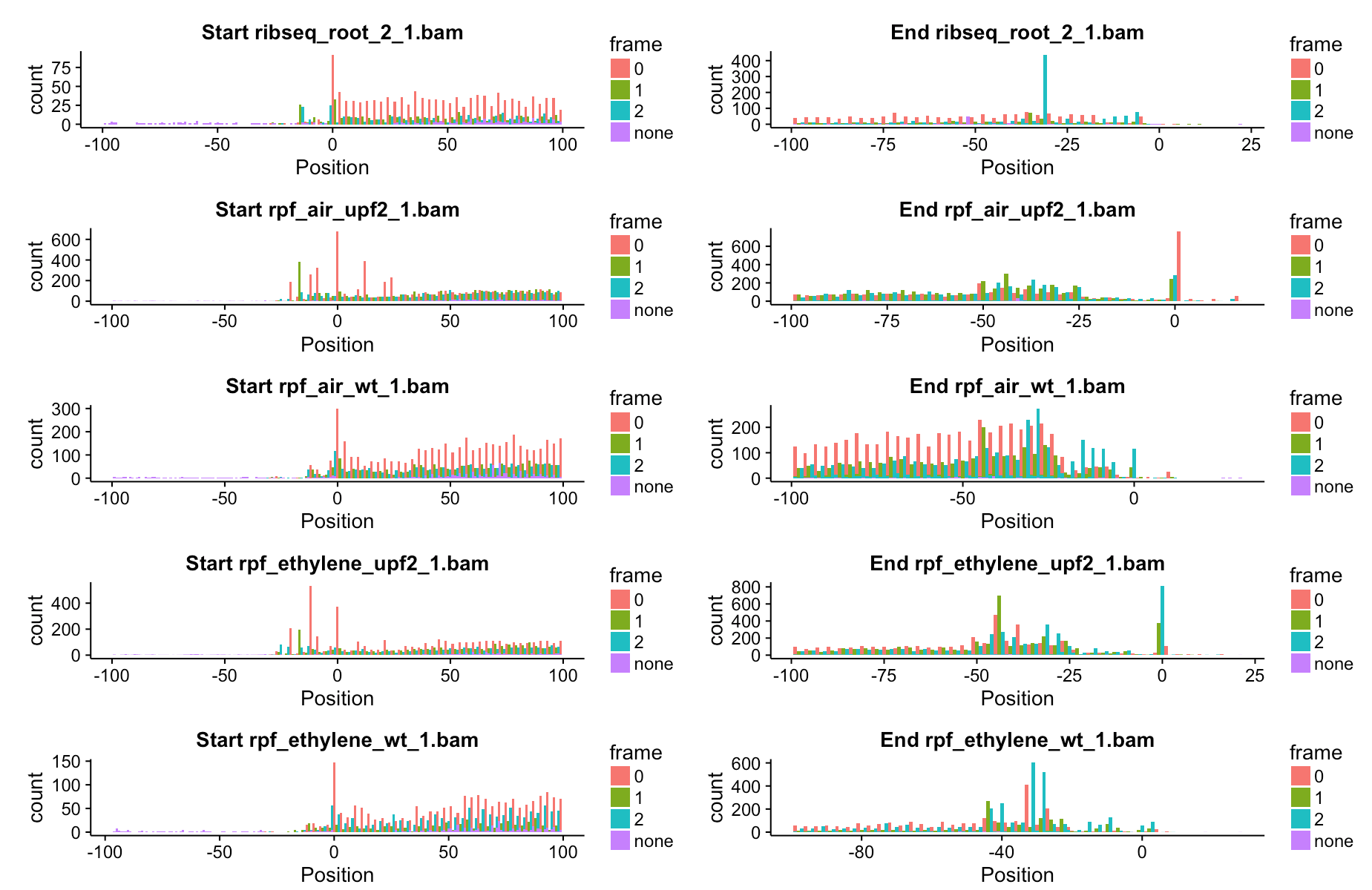


Figure S10. Meta-gene periodicity plots for the 5 samples. The plots on the left represent the aggregate read distributions around the coding sequence start sites, while those on the left are around the coding sequence end sites. The different colored bars represent reads in each of possible frames.

**Length periodicities**

The **Length Periodicity** tool can be used to further inspect the level of periodicity in the different samples. Figure S11 shows the output of this tool when the Read length parameter is set to 25-31 and the Choose how to separate groups parameter is set to ‘sample’. In this plot, each grouping of bars represents a separate sample, while each individual bar represents the reads of a certain length within that sample. The colors of each bar represent the relative abundances of reads of that length which are in positioned in each respective frame. As reads in frame 0 are considered in the major frame, bars with large red portions represent higher periodicity levels.


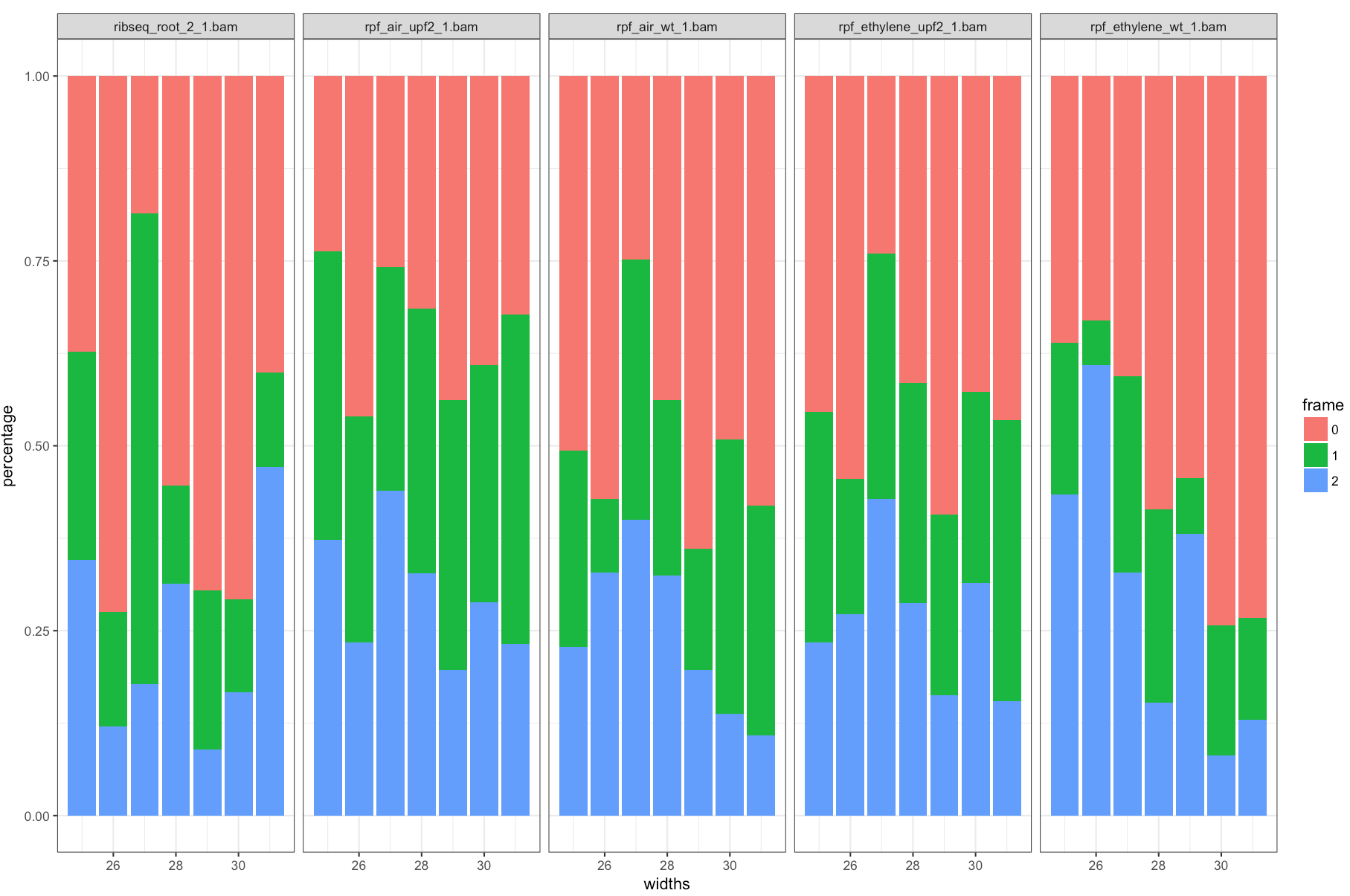


Figure S11. Length periodicity plot. Each grouping of bars represents a sample, while each bar represents all the reads of a certain read length within that sample. The colors show which of those read lengths are in each frame.

**Single gene read distributions**

If we are specifically interested in a certain gene, we can look at the distribution of reads in that gene using the **Gene Viewer** tool. If we select our gene (AT3G09260 in this example) in the Genes parameter field, set Choose the factor to separate lines to ‘frame’, and Choose the factor to separate plots to ‘sample’, then we produce the output shown in Figure S12. Exons are depicted as horizontal grey bars along the x-axis. In this case the colors represent the respective frames, and therefore the presence of a large number of red bars indicates a high periodicity level. Additionally, increased ‘dispersion’ in the coverage of the reads indicates high levels of complexity, while large isolated peaks in occupancy indicate low complexity, and might suggest read over-amplification issues.


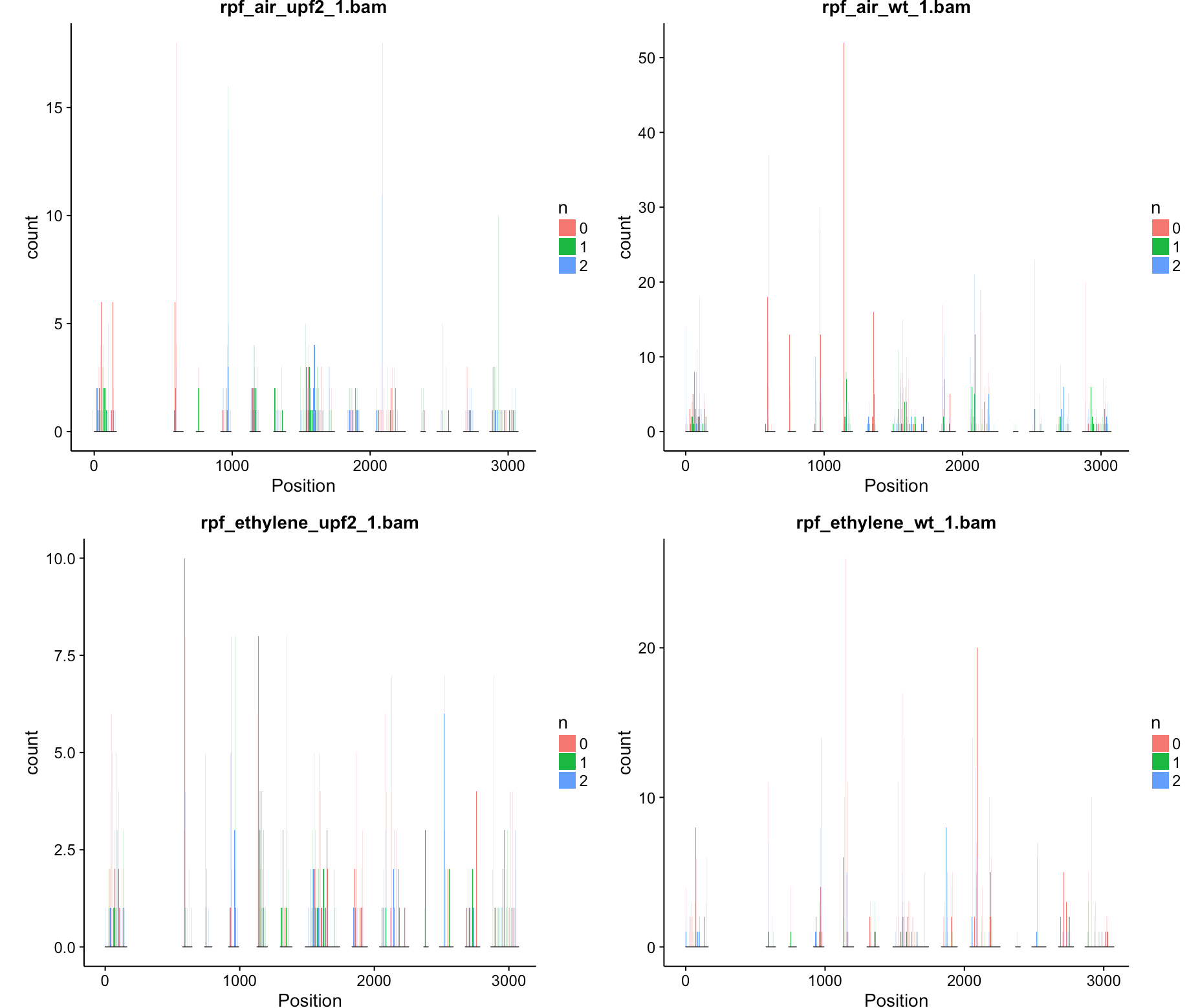


Figure S12. Read distributions about a single gene for 4 of the samples. The color of the bars indicates the frame of the reads. The gray bars above the x-axis indicate the location of the exons in the gene model.

**Report generation**

We can use the **Report Generation** tool to create a detailed report of this quality analysis for our collaborators. We can simply select which tools in the toolbar that we want included in our report, and an R markdown file will be generated which includes the output from each tool, as well as the list of parameters that were used to produce each graphic.

**Conclusion**

This concludes a basic Ribo-seq quality analysis using riboStreamR. As we have seen, there are many measures of sample quality that need to be assessed when performing Ribo-seq data analysis. The tools in riboStreamR are built specifically to investigate these numerous quality metrics, and are equipped with various options for creating customized output based on the user’s exact needs. This case study demonstrates that riboStreamR is a powerful fool for performing Ribo-seq data QC, and provides users with a flexible and user-friendly environment for exploring their data.

**References**

1. Merchante C, Brumos J, Yun J, Hu Q, Spencer KR, Enríquez P, Binder, BM, Heber S, Stepanova AN, Alonso JM. Gene-Specific Translation Regulation Mediated by the Hormone-Signaling Molecule EIN2. Cell. 2016;163:684-97
2. Hsu P, Calviello, Wu HY, Li FW, Rothfels C, Ohler U, Benfey P. Super-resolution ribosome profiling reveals unannotated translation events in Arabidopsis. Proceedings of the National Academy of Sciences. 1016;113. doi:10.1073/pnas.1614788113.
3. Trapnell C, Pachter L, Salzberg SL. TopHat: discovering splice junctions with RNA-Seq. Bioinformatics. 2009;25:1105–1111.
4. Berardini TZ, Reiser L, Li D, Mezheritsky Y, Muller R, Strait E, et al. The Arabidopsis information resource: making and mining the “gold standard” annotated reference plant genome. Genesis. 2015;53:474–485. doi: 10.1002/dvg.22877.
